# Co‐development of attention deficit hyperactivity disorder and autistic trait trajectories from childhood to early adulthood

**DOI:** 10.1111/jcpp.13851

**Published:** 2023-06-22

**Authors:** Amy Shakeshaft, Jon Heron, Rachel Blakey, Lucy Riglin, George Davey Smith, Evie Stergiakouli, Kate Tilling, Anita Thapar

**Affiliations:** ^1^ Division of Psychological Medicine and Clinical Neurosciences, Centre for Neuropsychiatric Genetics and Genomics Cardiff University Cardiff UK; ^2^ Wolfson Centre for Young People's Mental Health Cardiff University Cardiff UK; ^3^ Population Health Sciences and MRC Integrative Epidemiology Unit University of Bristol Bristol UK

**Keywords:** ADHD, Autism, ALSPAC, trajectories, longitudinal, genetic

## Abstract

**Background:**

Attention deficit hyperactivity disorder (ADHD) and autism, defined as traits or disorders, commonly co‐occur. Developmental trajectories of ADHD and autistic traits both show heterogeneity in onset and course, but little is known about how symptom trajectories co‐develop into adulthood.

**Methods:**

Using data from a population cohort, the Avon Longitudinal Study of Parents and Children, we examined correlations between ADHD and autistic traits across development, using the Social Communication Disorders Checklist and ADHD subscale of the Strengths and Difficulties Questionnaire. We modelled joint developmental trajectories of parent‐reported ADHD and autistic traits between 4 and 25 years, then characterised trajectory classes based on sociodemographic, perinatal, psychopathology, cognition and social functioning variables and tested for associations with neurodevelopmental/psychiatric polygenic scores (PGS).

**Results:**

Three classes of trajectories were identified; a typically developing majority with low‐stable ADHD‐autistic traits (87%), a male‐predominant subgroup with child/adolescent‐declining traits (6%) and a subgroup with late‐emerging traits (6%). ADHD‐autistic trait correlations were greatest in young adulthood for the two nontypically developing classes. There were higher rates of emotional and conduct problems, low IQ, childhood seizures and poor social functioning in the declining and late‐emerging classes compared to the low‐stable class. Emotional, conduct and peer problems were more prevalent during childhood in the childhood/adolescent‐declining class compared to other classes, but were more prevalent in young adulthood in the late‐emerging class. Neurodevelopmental/psychiatric PGS also differed: both nontypically developing classes showed elevated ADHD PGS compared to the low‐stable group, and the late‐emerging group additionally showed elevated schizophrenia PGS and decreased executive function PGS, whereas the declining group showed elevated broad depression PGS.

**Conclusions:**

Distinct patterns of ADHD‐autism co‐development are present across development in the general population, each with different characterising factors and genetic signatures as indexed by PGS.

## Introduction

Autism [or autism spectrum disorder (ASD)] and attention deficit hyperactivity disorder (ADHD) are both highly heritable, neurodevelopmental conditions that frequently co‐occur (Thapar, Cooper, & Rutter, 2017). Although conceptualised as binary diagnostic categories for clinical purposes, they also can be viewed as traits on a continuum, akin to hypertension and blood pressure (Lubke, Hudziak, Derks, Van Bijsterveldt, & Boomsma, 2009; Wittkopf et al., 2022). Risk factors for ADHD and autistic trait scores in the general population and clinical disorders overlap, with no clear‐cut threshold on trait measures beyond which adverse outcomes manifest (Lord, Elsabbagh, Baird, & Veenstra‐Vanderweele, 2018; Thapar & Cooper, 2016). ADHD trait scores in the general population and clinical ADHD share genetic influences (Larsson, Anckarsater, Råstam, Chang, & Lichtenstein, 2012; Stergiakouli et al., 2015; Taylor et al., 2019), and are highly genetically correlated (*r*_g_ = 0.97; Demontis et al., 2019). Similarly, evidence from twin and molecular genetic studies suggests the variation of autistic traits in the population (including variation in social communication and behaviours) and an autism diagnosis have a similar genetic aetiology (Colvert et al., 2015; Robinson et al., 2011, 2016; Taylor et al., 2019). These studies highlight that ADHD and autism can be viewed as traits as well as categorical diagnoses.

ADHD and autism share much in common. Both typically onset in early development, are more common in males, share heritability and share genetic aetiology (Lee et al., 2019; Thapar et al., 2017). They also have common risk factors such as preterm birth (Blencowe et al., 2013), and share common comorbidities, such as an increased risk of emotional problems in youth and later life (Thapar, Livingston, Eyre, & Riglin, 2023). The co‐occurrence of autism and ADHD is clinically important. Those with autism‐ADHD comorbidity show greater impairment, worse outcomes and pose a greater treatment challenge (Hartman, Geurts, Franke, Buitelaar, & Rommelse, 2016; Sokolova et al., 2017; Vaidya & Klein, 2022).

Although it is recognised that autism and ADHD can persist into adult life, both show developmental changes across the lifespan. To date, most studies of autism‐ADHD co‐occurrence focus on children, with fewer studies of co‐occurrence in later life (Hartman et al., 2016; St Pourcain et al., 2011). Previous work indicates autism‐ADHD co‐occurrence changes across the lifespan, with suggestions that co‐occurrence peaks in adolescence and early adulthood. This relationship may be stronger due to greater demands on executive function (EF, which is impaired in both autism and ADHD) and social skills during emerging adulthood (Hartman et al., 2016). The extent to which EF determines developmental patterns of ASD, ADHD and their co‐occurrence is debated (Hartman et al., 2016).

One hypothesis is that there are subgroups with different developmental changes in autism‐ADHD co‐occurrence (Hartman et al., 2016). When autism and ADHD are considered separately, longitudinal studies have identified multiple subgroups showing different patterns of change with age. In clinical samples, the majority show persistence of disorder, others show symptom decline with age or, for ADHD, symptoms can remit after childhood (Mccauley, Elias, & Lord, 2020; Orm, Øie, Fossum, Andersen, & Skogli, 2021). Population‐based studies show similar patterns, the majority in a consistently low ADHD or autism trait group, and subgroups with persistent and remitting patterns (Agnew‐Blais et al., 2016; Pender, Fearon, St Pourcain, Heron, & Mandy, 2021; Riglin et al., 2021, 2022). Multiple longitudinal studies have also observed late‐onset forms of ADHD, first manifesting in adolescence and adult life (Agnew‐Blais et al., 2016; Asherson & Agnew‐Blais, 2019; Caye et al., 2016; Cooper et al., 2018; Moffitt et al., 2015; Riglin et al., 2022; Sibley et al., 2021). The same may apply to autism, at least for social communication symptoms (Riglin, Wootton, et al., 2021), but late‐onset autism is not well established. These studies that have examined ADHD and autistic developmental trajectories separately observe that the different subgroups show different patterns of association with genetic risks, as indexed by polygenic scores (PGS; composite of common genetic risk variants) and other correlates.

Together, these findings highlight the developmental heterogeneity of autism and ADHD. We hypothesise that subgroups in the general population will show different developmental patterns of ADHD‐autistic trait co‐occurrence across childhood, adolescence and early adulthood. We further hypothesise that these subgroups will show different genetic signatures, indexed by neurodevelopmental and psychiatric PGS, vary in sociodemographic and perinatal features, psychopathology and child and adult functioning. We had two main aims:

1. Identify and characterise heterogeneity in ADHD‐autism co‐developmental trajectories from childhood to early adulthood (25 years).
2. Test for associations between different patterns of co‐development and neuropsychiatric PGS known to be associated with ADHD, autism and executive function.

## Methods

### Sample

The Avon Longitudinal Study of Parents and Children (ALSPAC) study is an ongoing longitudinal study which recruited pregnant woman in the Avon region of South‐West England, due to give birth between 1 April 1991 and 31 December 1992 (Boyd et al., 2013; Fraser et al., 2013; Northstone et al., 2019). The core sample consisted of 14,541 mothers and of these pregnancies, 13,988 children were alive at 1 year. Following the initial recruitment, an additional 913 children were recruited in three phases. Data were collected from families repeatedly and managed using REDCap (Harris et al., 2009). Ethical approval for the study was obtained from the ALSPAC Ethics and Law Committee and the Local Research Ethics Committees. Informed consent for the use of data collected via questionnaires and clinics was obtained from participants following the recommendations of the ALSPAC Ethics and Law Committee at the time. The study website contains details of all the data that are available through a fully searchable data dictionary and variable search tool (http://www.bristol.ac.uk/alspac/researchers/our-data/). Full details of the ALSPAC study can be found in Appendix S1. For families with multiple births, we included the oldest sibling.

### Measures and data collection

#### ADHD and autistic traits used to generate trajectories

ADHD traits were measured using the parent‐rated 5‐item Strengths and Difficulties Questionnaire (SDQ; Goodman, 1997) ADHD subscale. This was completed at ages 4, 7, 8, 9, 12, 13, 17 and 25 years. Additionally, the self‐rated questionnaire was completed by ALSPAC offspring at age 25. It is designed to measure hyperactive and inattentive symptoms, ranges from 0 to 10, and has been validated against a DSM‐5 diagnosis of ADHD in childhood and young adulthood (Riglin et al., 2021). Previous studies indicate SDQ scores have configural, metric, scalar and residual invariance across ages 7 to 16 years (Speyer, Auyeung, & Murray, 2022), as well as internal consistency of SDQ subscales in adolescents/young adults in a clinical setting (Brann, Lethbridge, & Mildred, 2018). For reference, between ages 4 and 17 years, scores between 0 and 5 are classified as low, scores of 6–7 as slightly raised, or scores of 8–10 as high ADHD symptoms (Goodman, 1997). At age 25, ≥4 is classified as raised ADHD symptoms for parent‐reports, and ≥5 as slightly raised and ≥6 as high ADHD symptoms in self‐reports (Riglin, Agha, et al., 2021). Questionnaires with >2 items missing were excluded. Details of imputation of missing items are given in Appendix S1.

Autistic traits were measured using the parent‐rated 10‐item Social Communication Disorder Checklist (SCDC; Skuse, Mandy, & Scourfield, 2005). This was completed at ages 7, 10, 13, 17 and 25 years, and has previously been shown to have acceptable measurement invariance across these ages (Riglin, Wootton, et al., 2021). It is designed to measure social reciprocity and verbal/nonverbal communication and has high sensitivity and specificity for an autism diagnosis (Bölte, Westerwald, Holtmann, Freitag, & Poustka, 2011). The questionnaire ranges from 0 to 24, with a score of ≥9 recommended as the cut‐point for pervasive developmental disorders, such as autism (Skuse et al., 2005). Details of imputation of missing items are given in Appendix S1.

#### Trajectory validators

Since trajectories were derived exclusively on parent‐rated ADHD (SDQ‐ADHD) and autistic (SCDC) traits to retain the consistency of informant across ages, we also report on self‐rated ADHD and autistic traits in young adulthood using the self‐rated SDQ‐ADHD subscale (detailed above) and the Autism Spectrum Quotient (AQ; Baron‐Cohen, Wheelwright, Skinner, Martin, & Clubley, 2001). The AQ has been validated as a measure of clinical autism in adults, and ranges from 0 to 50, with a score of ≥32 indicating likely autism (Baron‐Cohen et al., 2001). We also examined childhood ADHD diagnosis at age 7 using the 18‐item Development and Well‐Being Assessment (DAWBA) ADHD section (Goodman, Ford, Simmons, Gatward, & Meltzer, 2000). The DAWBA is a structured diagnostic interview that assesses the 18 DSM ADHD diagnostic symptoms and was completed by parents as a questionnaire. We also report on childhood autism diagnosis, which was reported by parents when offspring were approximately 9 years old.

### Characterising trajectory classes

To describe trajectory classes, we selected measures known to be associated with ADHD, autism or both.

#### Pre‐/perinatal variables

We investigated the frequency of preterm birth and low birth weight in each trajectory group. Preterm birth was defined as birth before 37 weeks' gestation. Low birth weight was defined as a birth weight of less than 2,500 g. Both measures were recorded at birth from obstetric records, ALSPAC measurements and birth notification records.

#### Socio‐demographic variables

We investigated sex, recorded at birth and family income in each trajectory group since previous evidence has shown links between socio‐economic status and ADHD (Michaëlsson et al., 2022; Russell, Ford, Williams, & Russell, 2016), and conflicting evidence for associations with autism depending on country (Kelly et al., 2017). Family income was reported by mothers when children were aged 11 as the average household income including social benefits each week on a 10‐point scale from <£120 to ≥£800. We binarised family income for the purpose of our analysis, classifying low family income as those with <£430 weekly income (the £430 to £479 strata was the median income category in the cohort).

#### Psychopathology, cognitive ability and childhood seizures

Parent‐rated emotional problems and conduct problems were measured using the emotional problems and conduct problem subscales of the SDQ (Goodman, 1997) at ages 7 (child), 13 (early adolescence) and 17 (late adolescence). Both parent and self‐ratings were obtained at 25 years (early adulthood). Each subscale score ranges from 0 to 10. A cut‐point of ≥4 indicates the presence of conduct problems and a cut‐point of ≥5 indicates the presence of emotional problems in childhood and adolescence (Goodman, 1997; Goodman et al., 2000). Additionally, self‐reported anxiety symptoms were measured at age 21 using the Generalised Anxiety Disorder Assessment‐7 (GAD‐7; Spitzer, Kroenke, Williams, & Lowe, 2006). Self‐reported mental well‐being was measured at age 23 using the Warwick‐Edinburgh Mental Well‐being Scale (WEMWBS; Tennant et al., 2007). Depressive symptoms were self‐reported at age 25 using the Short Mood and Feelings Questionnaire (SMFQ; Angold, Costello, Messer, & Pickles, 1995). Total symptom scores were calculated for each scale and we used recommended cut‐points for these measures to estimate probable depression (SMFQ ≥12; Eyre et al., 2021), generalised anxiety disorder (GAD‐7 ≥10; Spitzer et al., 2006) and poor mental well‐being (WEMWBS ≤40; Warwick Medical School, 2021).

Childhood IQ was measured in ALSPAC at age 8 using the Wechsler Intelligence Scale for Children – full IQ. An IQ score of <80 was considered low IQ. As epilepsy commonly co‐occurs with ADHD and autism and is considered a neurodevelopmental disorder, we included a measure of childhood seizures. The occurrence of seizures thought to be due to epilepsy up to the age of 11 was determined by asking parents of children in ALSPAC about any history of seizures and the cause of the seizure(s) at 18, 30, 42, 57, 69, 81, 103 months (approximately 2.5, 3.5, 4.8, 5.8, 6.8 and 8.6 years) and finally at 11 years.

#### Childhood and adult social functioning variables

The presence or absence of peer problems was measured using the peer problems subscale of SDQ (Goodman, 1997), parent‐rated at ages 7 (childhood), 13 (early adolescence) and 17 (late adolescence) and parent and self‐rated at 25 years (young adulthood). A cut‐point of ≥4 indicates the presence of peer problems in childhood and adolescence (Goodman, 1997). We investigated the frequency of those not in education, employment or training (NEET) in each trajectory group using a self‐report data supplied from ALSPAC participants at age 25. Alcohol abuse was measured using self‐report questionnaires in ALSPAC participants at age 22 using the DSM‐5 criteria for alcohol use disorder (AUD). We binarized the variable whereby we included anyone meeting mild, moderate or severe AUD criteria. Cannabis abuse was measured using the self‐report six‐item CAST [Cannabis Abuse Screening Test (Legleye, Piontek, & Kraus, 2011)] at age 22. As done previously in the ALSPAC cohort (Heron et al., 2013), we categorised those with a nonzero CAST score as ‘cannabis abuse’.

#### Genetic liability

We used PGS to index the common genetic liability for a variety of neurodevelopmental and psychiatric conditions. Summary statistics from the following studies were used to generate PGS: (a) ADHD – Demontis et al. (2019), (b) ASD – Grove et al. (2019), (c) schizophrenia – Trubetskoy et al. (2022), (d) bipolar disorder – Stahl et al. (2019), (e) broad depression – Howard et al. (2019), (f) major depression – Wray et al. (2018) and (g) executive function – Hatoum et al. (2020). These PGS were chosen since previous evidence suggests shared genetic aetiology between these disorders and ADHD/ASD (Larsson et al., 2013; Riglin et al., 2021). Additionally, the disorders indexed by these PGS are well‐established comorbidities or cognitive aspects of ADHD and autism (Thapar et al., 2023). Details of the generation of PGS and GWAS from which PGS were derived from, are supplied in Appendix S1 and Table S7. Each PGS was standardised to aid interpretation.

### Statistical analysis

#### Correlation between ADHD and autistic traits

Correlations between ADHD and autistic traits at approximately ages 7, 9/10, 13, 17 and 25 years were examined using Spearman's rank correlation. We used the subset of the cohort who had complete SDQ‐ADHD and SCDC questionnaires at age 7 (*n* = 7,156). To minimise attrition bias, we imputed any subsequent incomplete values using multiple imputation, using the mice package in R (Van Buuren & Groothuis‐Oudshoorn, 2011). SDQ‐ADHD values were imputed using the SDQ‐ADHD scores from all other timepoints, SCDC scores at age 7 and SCDC scores at the same timepoint and the prior timepoint as predictors (e.g. age 25 SDQ‐ADHD scores were imputed using all other SDQ‐ADHD scores and age 7, age 17 and age 25 SCDC scores). The converse was carried out for missing SCDC scores. Predictive mean matching was used to impute scores, generating 60 imputed datasets which were pooled and analysed.

#### Parallel‐process growth mixture modelling

We used parallel‐process growth mixture modelling (GMM) in MPlus, v.8.8 (Muthén & Muthén, 1998–2017) to model trajectories of parent‐reported ADHD and autistic traits between 4 and 25 years. Parallel‐process GMM aims to group individuals into classes based on patterns of change of two outcomes across multiple timepoints (note that the pattern of change for each outcome within a class is not required to be the same/similar in this model). Individuals in the same class are assumed to have the same growth curve, however within‐class heterogeneity is modelled, unlike latent class growth analysis. The average age in years at each questionnaire completion was used as the time metric for the model. Given the large gap between the last two time points and akin to previous ADHD and autism trajectory work in the same dataset (Riglin et al., 2022; Riglin, Wootton, et al., 2021), models were fitted with piecewise growth models with single intercept and two linear slope factors, one for ages 4–17 for ADHD traits and 7–17 for autistic traits, and another for ages 17 and 25 for ADHD and autistic traits. The variance of the second slope was fixed to zero to avoid nonidentification, as only two time points were included in these growth factors. The GMM, therefore, included one intercept, one slope for ages 4/7–17 and a second slope for ages 17–25 each for ADHD and autistic traits in parallel (see Figure S1). Residual variances were constrained to be time‐invariant for SCDC and SDQ‐ADHD, and between‐class covariances were also constrained due to difficulties running models with freed covariances. The primary sample included individuals for whom SCDC or SDQ‐ADHD data were available from at least one timepoint (*n* = 11,316). Models were fit using maximum likelihood estimator and full information maximum likelihood was used to handle missing data.

Starting with a single *k*‐class model, *k* + 1 solutions were fitted until the optimum model was reached, as determined primarily using Lo–Mendell–Rubin (LMR) tests, supported by Akaike information criterion (AIC), Bayesian information criterion (BIC), model entropy and smallest class size information. The LMR test quantifies whether a k‐class model substantially improves model fit enough to justify the additional parameters introduced compared to *k* − 1 class model. Lower values on AIC and BIC indicate better model fit and higher model entropy values indicate better distinguishability of classes when testing for association of auxiliary variables with trajectory classes. Once the optimal model was determined, the modelling was repeated with an increased number of random start values (*n* = 1,000) to ensure there were no problems with the local maxima and the correct solutions were obtained. Class sizes are reported based on the estimated model with *N*s rounded to the nearest integer.

Correlations between ADHD and autistic traits were examined again, as described previously, this time stratified by trajectory class membership. Differences between correlations at age 7 and age 25 were tested using the ‘cocor’ package in R (Diedenhofen & Musch, 2015), using Silver's method (Silver, Hittner, & May, 2004).

#### Characterising trajectory profiles

We next characterised trajectory classes across a range of variables known to be associated with ADHD and/or autism. We used the manual bias‐corrected three‐step approach (Heron, Croudace, Barker, & Tilling, 2015; Vermunt, 2010; Wickrama, Lee, O'Neal, & Lorenz, 2016), implemented in MPlus, to test for associations of trajectory classes with auxiliary binary variables. This method uses multinomial regression and accounts for measurement error in class assignment. Associations with auxiliary variables were conducted on all available data. Sample sizes for each analysis are supplied in Table S1. Additionally, we used DCAT in MPlus to estimate proportions ± standard error (SE) for binary variables (Table S2; Figure S2), and the BCH (Bolck–Croon–Hagenaars) method to estimate means ± SE and a Wald's Chi‐squared test for equality of means for continuous auxiliary variables (Asparouhov & Muthén, 2021; Bakk & Kuha, 2021) since other three‐step methods have been found to be unsuitable when modelling continuous dependent variables.

### Sensitivity analysis

We checked whether trajectory classes were similar in males and females separately, as reported in Appendix S1. Further, we repeated analysis of the characterising of trajectory classes using the original continuous variables which had been binarized for the main analysis [i.e. self‐report SDQ‐ADHD, AQ, gestational age, birthweight, all parent and self‐rated subscales of SDQ (emotional, conduct and peer problems), GAD7, SMFQ, WEMWBS and IQ]. A summary of all cut–points used for continuous variables is presented in Table S8. We report mean ± SE of each measure by trajectory class as well as testing for equality of means using BCH in MPlus (Table S4).

## Results

**Figure 1:**
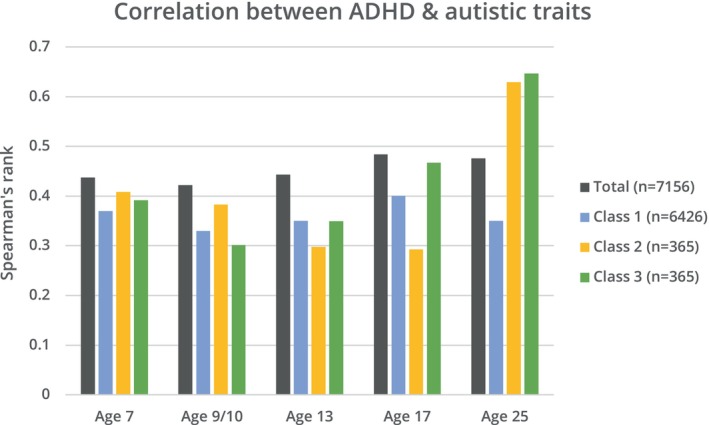
Correlation between autistic traits (measured using the SCDC) and ADHD traits (measured using SDQ‐ADHD subscale) at different timepoints throughout development, using Spearman's rank, in total cohort and stratified by trajectory class

The correlation between ADHD and autistic traits between 7 and 25 years in the whole cohort is shown in Figure 1 and the correlation matrix is presented in Table S3. Overall correlations were relatively stable across development (*r*_s_ = 0.4–0.5).

### Parallel‐process GMM

**Figure 2:**
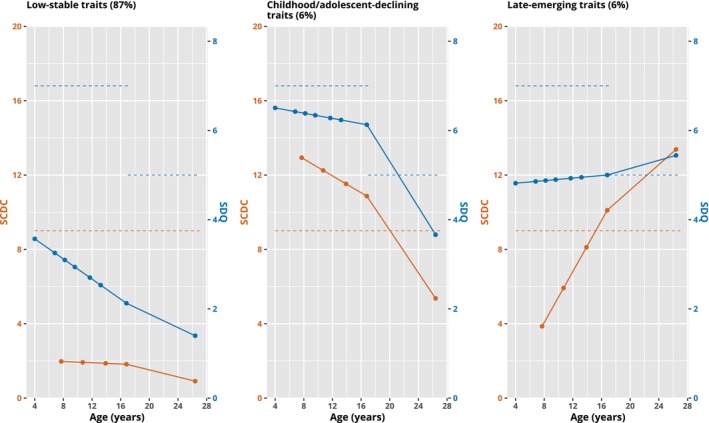
Mean trajectories of 3‐class GMM model. The blue line shows SDQ‐ADHD subscale mean values and orange line shows SCDC mean values. Recommended SCDC and SDQ‐ADHD cut‐points are shown with dashed lines of the same colours. Note the change in threshold for SDQ‐ADHD between childhood and late‐adolescence/adulthood

Results of GMM of SDQ‐ADHD and SCDC in 11,316 individuals showed a 3‐class model as the preferred fit (Table 1). While BIC, AIC, log‐likelihood values and LMR tests indicated a better fit with each added class (between 1 and 4), the 4‐class model included a small class (3%) below the recommended size of 5% (see Figure S3 for trajectories in the 4‐class model). An entropy value of 0.87 for the 3‐class model indicates high class separation. The chosen model included the following classes: Class 1, low‐stable ADHD‐autistic traits (87%) below the recommended cut‐points for SCDC and SDQ‐ADHD at all ages; Class 2, child/adolescent‐declining ADHD‐autistic traits (6%) above SCDC and SDQ‐ADHD cut‐points in childhood but falling below by late‐adolescence and Class 3, late‐emerging ADHD‐autistic traits (6%) below SCDC and SDQ‐ADHD cut‐points in childhood but rising above cut‐points by late‐adolescence (Figure 2).

**Table 1:** Model fit of growth mixture models with 1–4 classes

	1 Class	2 Class	3 Class	4 Class	5 Class
AIC	389,586	385,487	383,553	381,949	380,450
BIC	389,718	385,670	383,788	382,235	380,787
Entropy	–	0.90	0.87	0.85	0.83
Log‐likelihood	−194,775	−192,718	−191,745	−190,936	−190,179
LMR‐LRT *p*‐value	–	<0.00001	0.05	0.02	0.55
Class 1 *N* (%)	11,316 (100%)	10,386 (92%)	9,892 (87%)	9,192 (81%)	9,026 (80%)
Class 2 *N* (%)	–	930 (8%)	700 (6%)	1,193 (11%)	851 (8%)
Class 3 *N* (%)	–	–	724 (6%)	592 (5%)	637 (6%)
Class 4 *N* (%)	–	–	–	340 (3%)	553 (5%)
Class 5 *N* (%)	–	–	–	–	249 (2%)

AIC, Akaike information criterion; BIC, Bayesian information criterion; LMR‐LRT, Lo–Mendell–Rubin adjusted likelihood ratio test.

ADHD and autistic trait correlations increased with age when stratified by trajectory class (Figure 1). In the declining and late‐emerging symptom classes, the correlation increased from ages 7 to 25 [declining: *r*_s_ = 0.39 to *r*_s_ = 0.65 (*p* < .001); late‐emerging: *r*_s_ = 0.41 to *r*_s_ = 0.63 (*p* < .001)], whereas there was no change in the low‐stable class [*r*_s_ = 0.37 to *r*_s_ = 0.35 (*p* = .18)].

There was a higher proportion of individuals with an ADHD or autism diagnosis during childhood in the declining trait group, compared to both the late‐emerging and low‐stable groups (Table 2). The proportion of individuals with high ADHD and autistic traits in adulthood did not differ between the declining and late‐emerging classes, but both were higher than the low‐stable group (Table 2). For estimated proportions per class, see Table S2.

**Table 2:** Associations between additional autism and ADHD variables with trajectory classes

Autism/ADHD variables	Declining versus low‐stable			Late‐emerging versus low‐stable			Late‐emerging versus declining		
	OR	95% CI	*p*‐value	OR	95% CI	*p*‐value	OR	95% CI	*p*‐value
Autism diagnosis age 9 *(parent‐report)*	68.1	32.0, 145.0	<.001	2.6	0.3, 19.9	0.36	0.04	0.01, 0.25	.001
ADHD diagnosis age 7 *(DAWBA)*	210.8	45.3, 981.8	<.001	48.9	9.0, 266.4	<0.001	0.2	0.1, 0.4	<.001
High autistic traits age 25 *(self‐rated AQ)*	5.1	2.3, 11.5	<.001	5.2	2.4, 11.2	<0.001	1.0	0.4, 2.9	.98
High ADHD traits age 25 *(self‐rated SDQ‐ADHD)*	3.3	2.0, 5.3	<.001	3.3	2.1, 5.0	<0.001	1.0	0.5, 2.0	.97

### Characterising trajectory classes

**Figure 3:**
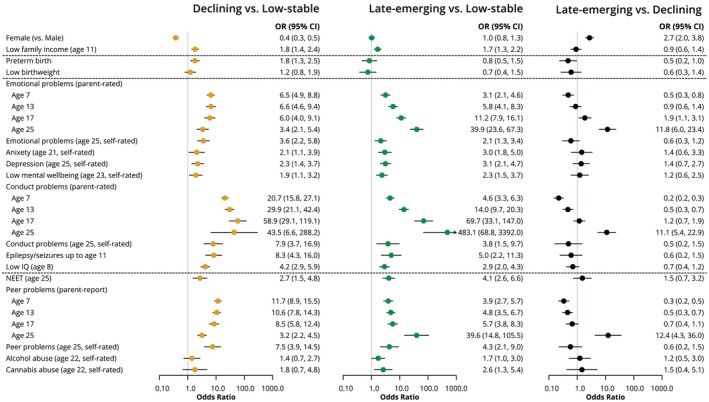
Results from bias‐adjusted three‐step associations of variables with trajectory classes

There are substantially more males in the child/adolescent‐declining group (73%) compared to the low‐stable [49%, OR = 0.38 (0.30, 0.47), *p* < .001] and late‐emerging [54%, OR = 0.37 (0.26, 0.51), *p* < .001] groups. The child/adolescent‐declining group also had the highest rate of preterm birth [8% vs. 5% in low‐stable (OR = 1.79 (1.26, 2.53), *p* = .001] and 5% in late‐emerging [OR = 2.11 (1.05, 4.23), *p* = .04) groups]. Low family income was also more common in both the declining and late‐emerging symptom groups than the low‐stable group (Figure 3).

#### Psychopathology, cognition and seizures

There were higher odds of emotional and conduct problems at all ages (parent and self‐rated), childhood epileptic seizures and low IQ in both the late‐emerging and child/adolescent‐declining groups, compared to the low‐stable symptom group (Figure 3; Table S2). Parent‐rated emotional and conduct problems were more common at age 7 in the child/adolescent‐declining group than other classes, however a higher proportion of individuals in the late‐emerging group had emotional and conduct problems at age 25, compared to the other classes (Figure 3).

#### Social functioning

There were higher odds of peer problems (parent and self‐rated) at all ages and NEET status at age 25 in both the late‐emerging and child/adolescent‐declining groups, compared to the low‐stable symptom group, and higher rates of substance abuse at age 22 in the late‐emerging group compared to the low‐stable group, but no difference compared to the declining symptom group (Figure 3; Table S2).

#### Genetic liability

**Figure 4:**
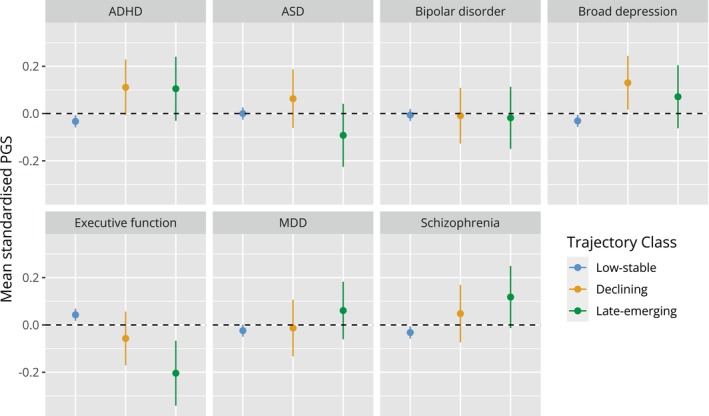
PGS for neurodevelopmental and psychiatric traits stratified by trajectory class. Means (±95% confidence intervals) for each trajectory class were calculated using the BCH method in MPlus. Note PGS have been *Z*‐score standardised to aid interpretation

Mean standardised PGS for each group are presented in Figure 4, with results from Wald's Chi‐squared test for equality of means presented in Table S4. PGS for all traits lie at a *Z*‐score of around 0 for the low‐stable symptoms group. In the childhood/adolescent‐declining class, ADHD (mean diff = 0.14, *p* = .02) and broad depression (mean diff = 0.16, *p* = .01; but not major depression) PGS were higher compared to the low‐stable group. In the late‐emerging class schizophrenia PGS was higher (mean diff = 0.15, *p* = .03) and executive function PGS was lower (mean diff = −0.24, *p* = .001) than the low‐stable symptom group, with weaker evidence for elevated ADHD PGS (mean diff = 0.14, *p* = .06).

### Sensitivity analysis

The best‐fit classes for sex‐stratified GMMs were less clear than in the overall analysis (see all models fits in Tables S5 and S6), however, both one (best fit for males) and two‐class (best fit for females) solutions (shown in Figures S4–S6) show overall similar trajectory patterns for ADHD and autistic traits between males and females.

Analysis of auxiliary variables associated with class membership using the original continuous variables in the overall cohort is shown in Table S4. This analysis showed similar patterns of association as when variables were binarized (as in Figure 3) for all variables except for anxiety, measured by the GAD7, for which the late‐emerging group had a higher self‐rated mean score at age 25 (mean = 7.4 ± SE 0.6) compared to the declining symptom group (mean = 4.9 ± SE 0.7, χ^2^ = 6.7, *p* = .01). In the main analysis, there was no difference in the proportion of individuals with probable generalised anxiety disorder between these two groups (Figure 3).

## Discussion

In this study, we investigated the co‐development of ADHD‐autistic traits from childhood to young adulthood in a large, prospective longitudinal birth cohort. Using parallel‐process GMM, we derived three distinct subgroups of hyperactive‐inattentive and social communication disorder symptoms: one with low‐stable ADHD‐autistic traits, reflecting the typically developing majority (87%), and two smaller subgroups both with 6% prevalence, one with child/adolescent‐declining ADHD‐autistic traits, and the other with late‐emerging ADHD‐autistic traits.

These trajectory classes are similar to those observed previously in this cohort when social communication and ADHD traits were examined separately (Riglin et al., 2022; Riglin, Wootton, et al., 2021). The patterns of ADHD development are also similar to those observed in other cohorts (Agnew‐Blais et al., 2016; Caye et al., 2016; Moffitt et al., 2015). In clinical settings, many autistic or ADHD individuals show persistence of symptoms and disorder. Given this is a population cohort with very few meeting diagnostic criteria for autism or ADHD, we suggest that those with persistent symptoms are likely represented by the declining symptom class, as a 4‐class solution (Figure S3) did identify a very small subgroup with persistent ADHD‐autistic traits who were mostly present in the declining symptom class in the 3‐class solution.

It now is well recognised that ADHD and autism commonly co‐occur and show a high level of correlation. Our findings suggest that autism and ADHD co‐develop in parallel from childhood to early adulthood. However, our findings also suggest that the patterns of developmental change maybe more complex than previously thought. While ADHD‐autistic trait correlations were stable for the total cohort, this was not the case in the nontypically developing subgroups (declining and late‐emerging). Here, symptom correlations increased with age, as previously hypothesised by Hartman et al. (2016). While most clinicians recognise the need to assess autism in children with ADHD, and vice versa, if our findings extend to clinical populations, they would indicate that autism may be missed in some adults with ADHD, and vice versa, since the relationship between symptoms increase over time and therefore was less clear‐cut earlier in development. If this finding is replicated in other studies, it may suggest that assessments of neurodevelopmental comorbidity should be repeated across the lifespan, not only at initial assessment in childhood.

The child/adolescent‐declining group and the late‐emerging group both showed higher rates of emotional and conduct problems, lower IQ, higher chance of childhood seizures and worse social functioning as indicated by peer problems and NEET status at age 25. However, some characteristics differentiated the two nontypically developing subgroups. First, the child/adolescent‐declining group showed the typical male predominance, concurring with previous literature that, in females, ADHD symptoms are less likely decline with age (Owens, Hinshaw, Lee, & Lahey, 2009). This group also had higher rates of preterm birth than the other classes. Second, while both nontypically developing groups had higher emotional and conduct problems throughout life than the low‐stable group, the late‐emerging group showed much higher rates of these psychopathologies and poor social functioning in early adulthood (age 25) than those with childhood/adolescent‐declining symptoms. This declining group rather showed higher rates of these problems during childhood and early adolescence (ages 7 and 13). This indicates that emotional and behavioural problems appear to be concurrent with ADHD‐autistic traits, such that problems peak when ADHD‐autistic traits peak. It is possible that this could be an artefact of parents being unable to distinguish different psychopathologies or difficulties, however, we saw little to no differences in self‐rated ADHD and autistic trait scores nor self‐reported psychopathology (e.g. emotional problems) between late‐emerging and declining groups.

Social functioning was also impaired in both nontypically developing groups and this was observed across all ages, with both groups showing peer problems across development and higher rates of being not in education or employment (NEET) in young adulthood. However, like emotional and conduct problems, peer problems were highest at ages when ADHD‐autistic traits peaked. Rates of substance abuse were also higher in the late‐emerging symptom group compared to the typically developing group.

Analysis of the genetic risk score profiles of these three subgroups, as indexed by PGS, suggested elevated ADHD genetic liability for the two nontypically developing subgroups, as well as decreased executive function PGS for both, compared to the typically developing majority. PGS for broad depression, but not major depressive disorder, was elevated in the childhood/adolescent‐declining subgroup only, whereas in the late‐emerging subgroup only, we saw elevated genetic liability for schizophrenia and lower PGS for executive function. This indicates that these two subgroups may have subtly different genetic architecture, with regard to common genetic risk variants (SNPs) associated with these psychiatric/neurodevelopmental disorders. It is interesting that the late‐emerging trait group has on average higher schizophrenia PGS, and schizophrenia typically first onsets after late‐adolescence/young adulthood. This may indicate that the timing of onset of different types of symptoms is associated with different genetic risk profiles. Conversely, higher depression (broadly defined) PGS are associated with declining ADHD‐autistic traits, yet depressive disorder more frequently onsets after adolescence rather than in childhood (Thapar, Eyre, Patel, & Brent, 2022). However, PGS associated with different psychiatric/neurodevelopmental disorders are highly correlated and nonspecific, that is, they cross diagnostic boundaries (Lee et al., 2019; Leppert et al., 2020). Thus, caution is needed in interpreting what different PGS profiles mean. Also, as PGS are weak indicators of genetic liability, and rare genetic variants also play an important role in neurodevelopmental disorders, these findings require further investigation.

These results should be viewed considering several limitations. First, the SCDC exclusively captures social and communication impairments, omitting the third phenotypic domain of autism: restricted and repetitive behaviours, including stereotyped movements, inflexible adherence to routines and sensory hypo/hyperreactivity (American Psychiatric Association, 2013). Therefore, it is possible that other domains of autism have different developmental trajectories and associations with ADHD symptom co‐occurrence and outcomes. Also, unlike the SDQ‐ADHD, which was first obtained in ALSPAC at age 4, the SCDC measure was first obtained at age 7 years, and therefore earlier measures could contribute to greater symptom variability and perhaps the emergence of different subgroups. Second, all measures used to create ADHD‐autistic trait trajectories are based on parent‐report, since self‐report was only available at age 25. Indeed, we see variability in SDQ subscale scores at age 25 depending on the reporter – for example, conduct and peer problems at age 25 are more common in the late‐emerging than the childhood/adolescent‐declining class when reported by parents, but there is little difference in self‐report, therefore indicating the possibility of rater effects, such that trajectory classes may only reflect symptoms as observed by parents. Additionally, since diagnoses through health records were not available, we relied on parental report of an autism diagnosis at age 9 and the self‐report AQ questionnaire at age 25 as trajectory class validators. While parent‐reported autism diagnosis does not include those diagnosed after 9 years, it remains useful in confirming that the questionnaire measure (SCDC) does identify clinical autism, as rates were higher in those with elevated ADHD‐autistic traits in childhood (child/adolescent declining class), compared to the other classes which do not. Third, as with many longitudinal studies, ALSPAC suffers from nonrandom attrition, with those at elevated risk of psychopathology, including those with high genetic risk for ADHD and depression, being more likely to drop‐out (Taylor et al., 2018). However, previous work on separate autism (Riglin, Wootton, et al., 2021) and ADHD (Riglin et al., 2022) trajectories in ALSPAC revealed similar patterns of results, when using different approaches to account for missing data. Finally, we must acknowledge the issue of multiple testing when looking at associations between auxiliary variables with ADHD‐autism trajectory classes which may increase the chance of false‐positive associations. Thus, replication in other population cohorts and clinical samples is required.

Nevertheless, our findings do suggest that developmental trajectories of ADHD and social communication traits appear to co‐develop in parallel, but that developmental patterns of emergence and decline, as well as their correlations, across the life‐course show heterogeneity. If shown in clinical populations, different patterns of co‐development will be an important consideration for clinicians following up autistic or ADHD children through to adult life.

## Conclusion

In summary, we observed heterogeneity in the joint trajectories of ADHD and autistic traits in the general population and differential patterns of symptom co‐occurrence from childhood to young adulthood. Trajectory subgroups indicate that autism and ADHD traits are reciprocal, whereby in most cases, they decline, persist or emerge together. Further, while both nontypically developing ADHD‐autism trajectory subgroups differ from the typically developing population in terms of sociodemographic and perinatal factors, psychopathology, child and adult function and genetic risk score profiles, there are some differences in the correlates and polygenic risk score profiles between the declining and late‐emerging subgroups.

**Key points**

- Autism and ADHD are both highly heritable neurodevelopmental conditions that frequently co‐occur and have heterogeneous developmental patterns. However, there is little known about patterns of ADHD‐autism co‐occurrence through development, particularly into adulthood.
- This work shows heterogeneous patterns of ADHD‐autistic trait co‐occurrence and development from childhood to young adulthood, characterised by different timings of symptom emergence, psychopathology, social functioning and genetic risk score profiles as indexed by PGS.
- These different patterns of symptom co‐development will be an important consideration for clinicians following up autistic or ADHD children through to adult life.

## Supporting information


**Table S1.** Sample size for association of variables with trajectory group analysis.
**Table S2.** Proportions of binary variables stratified by class (using DCAT in Mplus) with betas & ORs from bias‐adjusted 3‐step method in Mplus.
**Table S3.** Correlation between each SDQ‐ADHD and SCDC questionnaire timepoint.
**Table S4.** Mean ± SE of continuous variables stratified by trajectory class, with total and pairwise chi‐squared tests, using BCH method in Mplus.
**Table S5.** Female‐only GMM model fits.
**Table S6.** Male‐only GMM model fits.
**Table S7.** Details of GWAS summary statistics used for PGS generation.
**Table S8.** Definitions of binary variables where cut‐points of continuous variables have been used.

**Figure S1.** Basic path diagram depicting the parallel‐process growth mixture model used for analysis of ADHD‐autistic (ASD) traits.
**Figure S2.** Prevalence of associated features (±95% confidence intervals) by trajectory class.
**Figure S3.** Mean trajectories of 4‐class GMM model.
**Figure S4.** Female‐only 1‐class GMM symptom trajectory.
**Figure S5.** Female‐only 2‐class GMM symptom trajectories.
**Figure S6.** Male‐only 1‐class GMM symptom trajectory.
**Figure S7.** Male‐only 2‐class GMM symptom trajectories.
