# Supplementary material for: Co‐development of attention deficit hyperactivity disorder and autistic trait trajectories from childhood to early adulthood
Source: J Child Psychol Psychiatry. 2023 Jun 22;64(11):1596–607. doi: 10.1111/jcpp.13851 (PMC7615536; doi:10.1111/jcpp.13851)
Supplement: Supplementary file 1 [file JCPP-64-1596-s001.docx]

# Supporting Information

	

## Appendix S1.

#### ALSPAC study information

Pregnant women resident in Avon, UK with expected dates of delivery 1st April 1991 to 31st December 1992 were invited to take part in the study. The initial number of pregnancies enrolled is 14,541 (for these at least one questionnaire has been returned or a “Children in Focus” clinic had been attended by 19/07/99). Of these initial pregnancies, there was a total of 14,676 foetuses, resulting in 14,062 live births and 13,988 children who were alive at 1 year of age. 

When the oldest children were approximately 7 years of age, an attempt was made to bolster the initial sample with eligible cases who had failed to join the study originally. As a result, when considering variables collected from the age of seven onwards (and potentially abstracted from obstetric notes) there are data available for more than the 14,541 pregnancies mentioned above. The number of **new pregnancies** not in the initial sample (known as Phase I enrolment) that are currently represented on the built files and reflecting enrolment status at the age of 24 is 913 (456, 262 and 195 recruited during Phases II, III and IV respectively), resulting in an additional 913 children being enrolled. The phases of enrolment are described in more detail in the cohort profile paper and its update (see footnote 4 below). The total sample size for analyses using any data collected after the age of seven is therefore 15,454 pregnancies, resulting in 15,589 foetuses. Of these 14,901 were **alive at 1 year of age**. 

A 10% sample of the ALSPAC cohort, known as the **Children in Focus (CiF) group**, attended clinics at the University of Bristol at various time intervals between 4 to 61 months of age. The CiF group were chosen at random from the last 6 months of ALSPAC births (1432 families attended at least one clinic). Excluded were those mothers who had moved out of the area or were lost to follow-up, and those partaking in another study of infant development in Avon.

Study data were collected and managed using REDCap electronic data capture tools hosted at the University of Bristol (Harris et al., 2009). REDCap (Research Electronic Data Capture) is a secure, web-based software platform designed to support data capture for research studies. Ethical approval for the study was obtained from the ALSPAC Ethics and Law Committee and the Local Research Ethics Committees. Informed consent for the use of data collected via questionnaires and clinics was obtained from participants following the recommendations of the ALSPAC Ethics and Law Committee at the time.

The study website contains details of all the data that is available through a fully searchable data dictionary and variable search tool ([http://www.bristol.ac.uk/alspac/researchers/our-data](http://www.bristol.ac.uk/alspac/researchers/our-data)/).

#### Imputation of missing questionnaire items

SDQ subscale (ADHD, conduct problems, peer problems, emotional problems) questionnaires were discarded where >2 items were missing. In line with recommendations ([www.sdqinfo.org](http://www.sdqinfo.org)) total subscale scores were derived using mean imputation for those with (≤2) of items missing.

For the SCDC, questionnaires with >6 items missing were discarded. For those with ≤6 items missing, total scores were scaled by a factor of 12 / (12 – no. missing items):

For the self-rated Autism Spectrum quotient (AQ), questionnaires with >10% of items missing were not used. Where between 1-5 items were missing, items were corrected as follows: corrected score=raw score x (50 / (50 – no. missing items).

#### Calculation of Polygenic Scores

Polygenic scores (PRS) were derived for 9,912 ALSPAC children who were genotyped using the Illumina HumanHap500-quad genotyping array. Individuals were excluded based on gender mismatches, minimal or excessive heterozygosity, genotype missingness (>3%), insufficient sample replication (IBD <0.8), non-European ancestry (assessed by multidimensional scaling analysis and compared with Hapmap II) and cryptic relatedness (IBD > 0.1). SNPs were excluded based on minor allele frequency (<1%), call rate (<95%) or evidence for violations of Hardy-Weinberg equilibrium (P < 5E-7). Imputation was conducted by the ALSPAC team using Impute V2.2.2 against the 1000 genomes reference panel (Phase 1, Version 3: all polymorphic SNPs excluding singletons), using all 2,186 reference haplotypes (including non-Europeans). Best guess SNPs were subsequently filtered based on minor allele frequency (<1%) and imputation quality (INFO<0.8).

Genome-wide association study (GWAS) summary statistics were filtered to remove SNPs that were palindromic, insertions/deletions, non-autosomal, INFO score <0.8, missing in N>1 study and duplicates ([https://github.com/CardiffMRCPathfinder/summaRygwasqc](https://github.com/CardiffMRCPathfinder/summaRygwasqc)). PGS were generated for individuals in ALSPAC as the number of disorder risk alleles – defined using the GWAS summary statistics - weighted by effect size, using PRSice version 1.25 (Euesden, Lewis & O'Reilly, 2015) for all PGS except Schizophrenia (Trubetskoy et al., 2022) which used the same methods but in PRSice version 2.35 (Choi & O'Reilly, 2019). SNPs were clumped with an R^2^ threshold of 0.1 and a distance threshold of 1000kb and excluding the extended major histocompatibility complex (MHC; chromosome 6: 26-33Mb) due to the high linkage disequilibrium (LD) within this region. PRS were generated using GWAS outlined in Table S7, with risk alleles defined as those associated with case-status at p<0.05. ALSPAC was not included in any GWAS. 

#### Sex-stratified parallel-process growth mixture models

We performed sex-stratified parallel-process GMM, using the same methodology as in the overall models. Model fits are shown in Table S5 and S6. The best-fit classes for both male and female GMMs were less clear than in the overall analysis. For both males and females, model-fit (AIC, BIC and Entropy, shown in Table S5 & S6) continued to improve with the addition of more classes. However, in females the LMR p-value indicated the addition of a third class did not significantly improve the fit compared to the 2-class model (p=0.08). In males, the addition of a second class did improve model-fit compared to the single class trajectory model, however not significantly (p=0.10). Single and two-class trajectories for sex-stratified models are plotted in Figures S4-S7, where we see very similar patterns in males and females. 

## Supplementary Tables

Table S1 - Sample size for association of variables with trajectory group analysis.

Table S2 - Proportions of binary variables stratified by class (using DCAT in Mplus) with betas & ORs from bias-adjusted 3-step method in Mplus. 

Table S3 – Correlation between each SDQ-ADHD and SCDC questionnaire timepoint.

Table S4 - Mean ± SE of continuous variables stratified by trajectory class, with total and pairwise chi-squared tests, using BCH method in Mplus.

Table S5 - Female only GMM model fits.

Table S6 - Male only GMM model fits.

Table S7 - Details of GWAS summary statistics used for PGS generation.

Table S8 - Definitions of binary variables where cut-points of continuous variables have been used.

***See Supplementary_table.xls file for tables.***

## Supplementary Figures

 

Figure S1 – Basic path diagram depicting the parallel process growth mixture model used for analysis of ADHD-autistic (ASD) traits.

Figure S2 - Prevalence of associated features (±95% confidence intervals) by trajectory class. Proportions ±95% confidence intervals calculated using DCAT in Mplus, with the exception of variables with * which were estimated manually using most likely latent class membership.

Figure S3 – Mean trajectories of 4-class GMM model. The blue line shows SDQ-ADHD subscale mean values and orange line shows SCDC mean values. Note trajectory classes 1-3 are similar to the 3-class model, with a low-stable, declining and late-emerging group. The fourth class shows persistent-high autistic and ADHD traits. For class sizes and model fit see Table 1 in the main text. 

Figure S4 – Female-only 1-class GMM symptom trajectory. 

Figure S5 – Female-only 2-class GMM symptom trajectories. 

Figure S6 – Male-only 1-class GMM symptom trajectory.

Figure S7 – Male-only 2-class GMM symptom trajectories.

#### References

Choi, S. W.  &  O'reilly, P. F. (2019). Prsice-2: Polygenic risk score software for biobank-scale data. *Gigascience,* 8.

Euesden, J.,  Lewis, C. M.  &  O'reilly, P. F. (2015). Prsice: Polygenic risk score software. *Bioinformatics,* 31**,** 1466-1468.

Harris, P. A.,  Taylor, R.,  Thielke, R.,  Payne, J.,  Gonzalez, N.  &  Conde, J. G. (2009). Research electronic data capture (redcap)--a metadata-driven methodology and workflow process for providing translational research informatics support. *J Biomed Inform,* 42**,** 377-381.

Trubetskoy, V.,  Pardiñas, A. F.,  Qi, T.,  Panagiotaropoulou, G.,  Awasthi, S.,  Bigdeli, T. B.,  Bryois, J.,  Chen, C.-Y.,  Dennison, C. A.,  Hall, L. S.,  Lam, M.,  Watanabe, K.,  Frei, O.,  Ge, T.,  Harwood, J. C.,  Koopmans, F.,  Magnusson, S.,  Richards, A. L.,  Sidorenko, J.,  Wu, Y.,  Zeng, J.,  Grove, J.,  Kim, M.,  Li, Z.,  Voloudakis, G.,  Zhang, W.,  Adams, M.,  Agartz, I.,  Atkinson, E. G.,  Agerbo, E.,  Al Eissa, M.,  Albus, M.,  Alexander, M.,  Alizadeh, B. Z.,  Alptekin, K.,  Als, T. D.,  Amin, F.,  Arolt, V.,  Arrojo, M.,  Athanasiu, L.,  Azevedo, M. H.,  Bacanu, S. A.,  Bass, N. J.,  Begemann, M.,  Belliveau, R. A.,  Bene, J.,  Benyamin, B.,  Bergen, S. E.,  Blasi, G.,  Bobes, J.,  Bonassi, S.,  Braun, A.,  Bressan, R. A.,  Bromet, E. J.,  Bruggeman, R.,  Buckley, P. F.,  Buckner, R. L.,  Bybjerg-Grauholm, J.,  Cahn, W.,  Cairns, M. J.,  Calkins, M. E.,  Carr, V. J.,  Castle, D.,  Catts, S. V.,  Chambert, K. D.,  Chan, R. C. K.,  Chaumette, B.,  Cheng, W.,  Cheung, E. F. C.,  Chong, S. A.,  Cohen, D.,  Consoli, A.,  Cordeiro, Q.,  Costas, J.,  Curtis, C.,  Davidson, M.,  Davis, K. L.,  De Haan, L.,  Degenhardt, F.,  Delisi, L. E.,  Demontis, D.,  Dickerson, F.,  Dikeos, D.,  Dinan, T.,  Djurovic, S.,  Duan, J.,  Ducci, G.,  Dudbridge, F.,  Eriksson, J. G.,  Fañanás, L.,  Faraone, S. V.,  Fiorentino, A.,  Forstner, A.,  Frank, J.,  Freimer, N. B.,  Fromer, M.,  Frustaci, A.,  Gadelha, A.,  Genovese, G.,  Gershon, E. S.,  Giannitelli, M.,  Giegling, I.,  Giusti-Rodríguez, P.,  Godard, S.,  Goldstein, J. I.,  González Peñas, J.,  González-Pinto, A.,  Gopal, S.,  Gratten, J.,  Green, M. F.,  Greenwood, T. A.,  Guillin, O.,  Gülöksüz, S.,  Gur, R. E.,  Gur, R. C.,  Gutiérrez, B.,  Hahn, E.,  Hakonarson, H.,  Haroutunian, V.,  Hartmann, A. M.,  Harvey, C.,  Hayward, C.,  Henskens, F. A.,  Herms, S.,  Hoffmann, P.,  Howrigan, D. P.,  Ikeda, M.,  Iyegbe, C.,  Joa, I.,  Julià, A.,  Kähler, A. K.,  Kam-Thong, T.,  Kamatani, Y.,  Karachanak-Yankova, S.,  Kebir, O.,  Keller, M. C.,  Kelly, B. J.,  Khrunin, A.,  Kim, S.-W.,  Klovins, J.,  Kondratiev, N.,  Konte, B.,  Kraft, J.,  Kubo, M.,  Kučinskas, V.,  Kučinskiene, Z. A.,  Kusumawardhani, A.,  Kuzelova-Ptackova, H.,  Landi, S.,  Lazzeroni, L. C.,  Lee, P. H.,  Legge, S. E.,  Lehrer, D. S.,  Lencer, R.,  Lerer, B.,  Li, M.,  Lieberman, J.,  Light, G. A.,  Limborska, S.,  Liu, C.-M.,  Lönnqvist, J.,  Loughland, C. M.,  Lubinski, J.,  Luykx, J. J.,  Lynham, A.,  Macek, M.,  Mackinnon, A.,  Magnusson, P. K. E.,  Maher, B. S.,  Maier, W.,  Malaspina, D.,  Mallet, J.,  Marder, S. R.,  Marsal, S.,  Martin, A. R.,  Martorell, L.,  Mattheisen, M.,  Mccarley, R. W.,  Mcdonald, C.,  Mcgrath, J. J.,  Medeiros, H.,  Meier, S.,  Melegh, B.,  Melle, I.,  Mesholam-Gately, R. I.,  Metspalu, A.,  Michie, P. T.,  Milani, L.,  Milanova, V.,  Mitjans, M.,  Molden, E.,  Molina, E.,  Molto, M. D.,  Mondelli, V.,  Moreno, C.,  Morley, C. P.,  Muntané, G.,  Murphy, K. C.,  Myin-Germeys, I.,  Nenadić, I.,  Nestadt, G.,  Nikitina-Zake, L.,  Noto, C.,  Nuechterlein, K. H.,  O’brien, N. L.,  O’neill, F. A.,  Oh, S.-Y.,  Olincy, A.,  Ota, V. K.,  Pantelis, C.,  Papadimitriou, G. N.,  Parellada, M.,  Paunio, T.,  Pellegrino, R.,  Periyasamy, S.,  Perkins, D. O.,  Pfuhlmann, B.,  Pietiläinen, O.,  Pimm, J.,  Porteous, D.,  Powell, J.,  Quattrone, D.,  Quested, D.,  Radant, A. D.,  Rampino, A.,  Rapaport, M. H.,  Rautanen, A.,  Reichenberg, A.,  Roe, C.,  Roffman, J. L.,  Roth, J.,  Rothermundt, M.,  Rutten, B. P. F.,  Saker-Delye, S.,  Salomaa, V.,  Sanjuan, J.,  Santoro, M. L.,  Savitz, A.,  Schall, U.,  Scott, R. J.,  Seidman, L. J.,  Sharp, S. I.,  Shi, J.,  Siever, L. J.,  Sigurdsson, E.,  Sim, K.,  Skarabis, N.,  Slominsky, P.,  So, H.-C.,  Sobell, J. L.,  Söderman, E.,  Stain, H. J.,  Steen, N. E.,  Steixner-Kumar, A. A.,  Stögmann, E.,  Stone, W. S.,  Straub, R. E.,  Streit, F.,  Strengman, E.,  Stroup, T. S.,  Subramaniam, M.,  Sugar, C. A.,  Suvisaari, J.,  Svrakic, D. M.,  Swerdlow, N. R.,  Szatkiewicz, J. P.,  Ta, T. M. T.,  Takahashi, A.,  Terao, C.,  Thibaut, F.,  Toncheva, D.,  Tooney, P. A.,  Torretta, S.,  Tosato, S.,  Tura, G. B.,  Turetsky, B. I.,  Üçok, A.,  Vaaler, A.,  Van Amelsvoort, T.,  Van Winkel, R.,  Veijola, J.,  Waddington, J.,  Walter, H.,  Waterreus, A.,  Webb, B. T.,  Weiser, M.,  Williams, N. M.,  Witt, S. H.,  Wormley, B. K.,  Wu, J. Q.,  Xu, Z.,  Yolken, R.,  Zai, C. C.,  Zhou, W.,  Zhu, F.,  Zimprich, F.,  Atbaşoğlu, E. C.,  Ayub, M.,  Benner, C.  &  Bertolino, A. (2022). Mapping genomic loci implicates genes and synaptic biology in schizophrenia. *Nature,* 604**,** 502-508.
